# Colonic Epithelial Permeability to Ions Is Restored after Vedolizumab Treatment and May Predict Clinical Response in Inflammatory Bowel Disease Patients

**DOI:** 10.3390/ijms25115817

**Published:** 2024-05-27

**Authors:** Michele Cicala, Manuele Gori, Paola Balestrieri, Annamaria Altomare, Alessandro Tullio, Simone Di Cola, Sander Dejongh, Maria Giovanna Graziani, Cristiano Pagnini, Simone Carotti, Giuseppe Perrone, Mentore Ribolsi, Marcello Fiorani, Michele P. L. Guarino, Ricard Farré

**Affiliations:** 1 Gastroenterology Unit, Fondazione Policlinico Universitario Campus Bio-Medico and Università Campus Bio-Medico di Roma, 00128 Rome, Italy; m.cicala@policlinicocampus.it (M.C.); p.balestrieri@policlinicocampus.it (P.B.); a.altomare@policlinicocampus.it (A.A.); a.tullio@policlinicocampus.it (A.T.); s.dicola@policlinicocampus.it (S.D.C.); m.ribolsi@policlinicocampus.it (M.R.); marcellofiorani94@gmail.com (M.F.); m.guarino@policlinicocampus.it (M.P.L.G.); 2 Institute of Biochemistry and Cell Biology (IBBC), National Research Council (CNR), International Campus “A. Buzzati-Traverso”, Via E. Ramarini 32, Monterotondo Scalo, 00015 Rome, Italy; 3 Translational Research Center for Gastrointestinal Disorders (TARGID), KU Leuven, 3000 Leuven, Belgium; sander.dejongh@kuleuven.be (S.D.); ricard.farre@kuleuven.be (R.F.); 4 Laboratory of Nephrology and Renal Transplantation, KU Leuven, 3000 Leuven, Belgium; 5 Department of Gastroenterology and Digestive Endoscopy, S. Giovanni Addolorata Hospital, 00184 Rome, Italy; mggraziani@hsangiovanni.roma.it (M.G.G.); cpagnini@hsangiovanni.roma.it (C.P.); 6 Microscopic and Ultrastructural Anatomy Research Unit Medicine and Surgery, Università Campus Bio-Medico di Roma, 00128 Rome, Italy; s.carotti@policlinicocampus.it; 7 Predictive Molecular Diagnostics, Fondazione Policlinico Universitario Campus Bio-Medico, 00128 Rome, Italy; 8 Anatomical Pathology Operative Research Unit, Fondazione Policlinico Universitario Campus Bio-Medico, 00128 Rome, Italy; g.perrone@policlinicocampus.it; 9 Centro de Investigación Biomédica en Red de Enfermedades Hepáticas y Digestivas (CIBEREHD), 28029 Madrid, Spain

**Keywords:** intestinal barrier function, colonic epithelial permeability, integrin-receptor antagonist, inflammatory bowel disease, transepithelial electrical resistance, Ussing chambers

## Abstract

Vedolizumab (VDZ) is used for treating inflammatory bowel disease (IBD) patients. A study investigating colonic epithelial barrier function ex vivo following VDZ is lacking. This work aims to evaluate ex vivo the colonic epithelial barrier function in IBD patients at baseline and during VDZ treatment, and to investigate the relationships between barrier function and clinical parameters. Colonic specimens were obtained from 23 IBD patients before, and at 24 and 52 weeks after VDZ treatment, and from 26 healthy volunteers (HV). Transepithelial electrical resistance (TEER, permeability to ions) and paracellular permeability were measured in Ussing chambers. IBD patients showed increased epithelial permeability to ions (TEER, 13.80 ± 1.04 Ω × cm^2^ vs. HV 20.70 ± 1.52 Ω × cm^2^, *p* < 0.001) without changes in paracellular permeability of a 4 kDa probe. VDZ increased TEER (18.09 ± 1.44 Ω × cm^2^, *p* < 0.001) after 52 weeks. A clinical response was observed in 58% and 25% of patients at week 24, and in 62% and 50% at week 52, in ulcerative colitis and Crohn’s disease, respectively. Clinical and endoscopic scores were strongly associated with TEER. TEER < 14.65 Ω × cm^2^ predicted response to VDZ (OR 11; CI 2–59). VDZ reduces the increased permeability to ions observed in the colonic epithelium of IBD patients before treatment, in parallel to a clinical, histological (inflammatory infiltrate), and endoscopic improvement. A low TEER predicts clinical response to VDZ therapy.

## 1. Introduction

Inflammatory bowel diseases (IBDs), such as Crohn’s disease (CD) and ulcerative colitis (UC), are characterized by over-activation and dysregulation of the immune system and a relapsing-remitting presentation. It has been hypothesized that the interaction between the genome, the exposome, the microbiome, and the immunome can drive the pathogenesis of these disorders [1]. This complex interaction involves the gut barrier, capable of defending the host from harmful agents and simultaneously allowing for the passage of nutrients and solutes [2].

Intestinal barrier dysfunction has been observed in many intestinal and extra-intestinal diseases [3]. In IBD, the expression of the tight junction (TJs) proteins is altered [4,5,6,7], and growing evidence suggests that the gut barrier dysfunction can be a main factor involved in the pathogenesis [8,9,10].

Different studies in Ussing chambers, with small sample sizes, have confirmed that the colonic inflamed mucosa of IBD patients has an impaired epithelial barrier function at different levels, with heterogeneous findings. Increased permeability to ions has been shown in some studies [7,11,12], but others failed to show the same [13,14,15]. Schmitz et al. found that the increased permeability to ions was concomitant with increased paracellular permeability in the UC-inflamed colon [7], findings later confirmed in patients during a clinical remission [16].

Only a few studies, including few subjects, have addressed whether the epithelial barrier of the non-inflamed colonic mucosa of IBD patients in remission is altered [14,16,17]. These results are discrepant, and they could be due to the deep remission of the disease, with no relapse for several years [14].

IBD is characterized by an impaired immune response, giving rise to gut chronic inflammation associated with an increased expression of mucosal vascular addressin cell adhesion molecule 1 (MADCAM1) and infiltrating CD4+ and α4β7 integrin+ T cells [18]. Hence, a novel therapeutic strategy for UC and CD patients, vedolizumab (VDZ), aims at targeting gut leukocyte trafficking by selectively neutralizing α4β7 integrin and preventing its interaction with the MADCAM1 in the endothelium of blood vessels [19]. VDZ has been used since 2014, showing high efficacy and safety profiles, as well as a highly favorable risk–benefit ratio [20].

Currently, there is no available data on the possible effects of VDZ on the inflamed and non-inflamed colonic epithelial barrier function in terms of epithelial permeability. Therefore, we hypothesized that VDZ could improve inflammation and intestinal barrier function in IBD patients. Hence, the first aim was to assess the presence of an altered epithelial barrier function ex vivo in macroscopically minimally inflamed epithelium of IBD patients by using colonic biopsies. Our second aim was to evaluate the possible effect of VDZ on the epithelial barrier integrity. Thus, we evaluated ex vivo, in a prospective study, the colonic epithelial barrier function in IBD patients at baseline and following 24 and 52 weeks of treatment. Moreover, the relationship between clinical parameters and alterations of the colonic barrier function was investigated.

## 2. Results

### 2.1. Study Population

**Figure 1:**
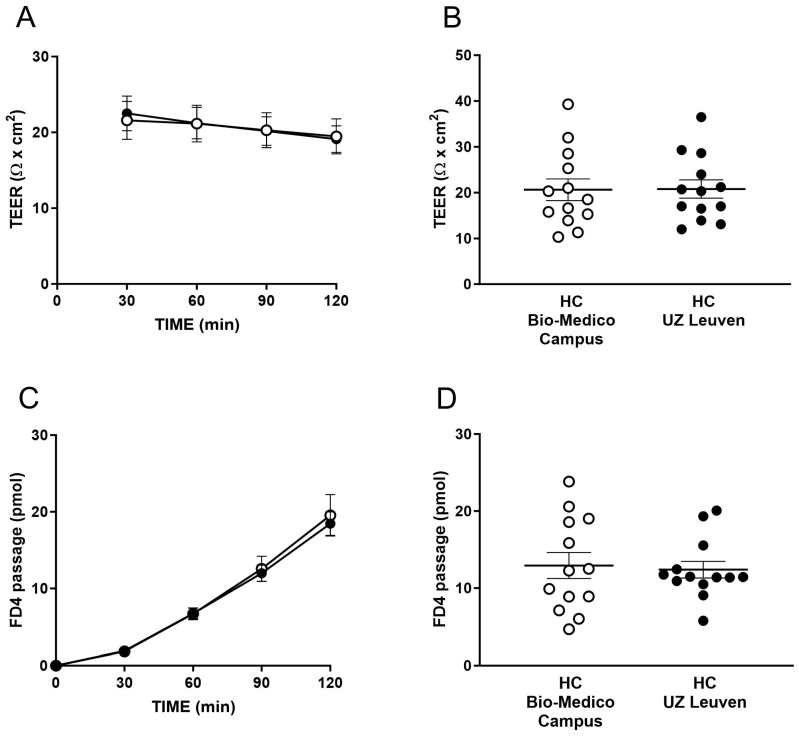
Epithelial barrier function in the two cohorts of healthy controls (HC). Transepithelial electrical resistance (TEER) at the time points 30, 60, 90 and 120 min (**A**), and as the average over 30–120 min (**B**). Paracellular passage of fluorescein isothiocyanate-dextran (FD4) at the time points 30, 60, 90 and 120 min (**C**), and as the average over 60–120 min (**D**). (**A**–**D**) Data are mean ± standard error of the mean (SEM), n = 13 subjects per group; experiments were performed using four biopsies/subject. Unpaired *t*-test in (**B**,**D**) and two-way ANOVA in (**A**,**C**) were used for comparison between groups.

Out of 23 total patients treated with VDZ, 15 (65.2%) were refractory to anti-TNFα, and 8 (34.8%) were biological-naïve patients. Baseline characteristics of patients stratified according to the type of disease (CD vs. UC) compared to HC are in Table 1. TEER and the passage of FD4 at baseline from four HC and one UC patient could not be measured due to the insufficient size or incorrect placement of the biopsies. The TEER and passage of FD4 were comparable between the two cohorts of HC included in the present study (Figure 1).

### 2.2. The Colonic Epithelium of IBD Patients Presented an Increased Permeability to Ions

**Figure 2:**
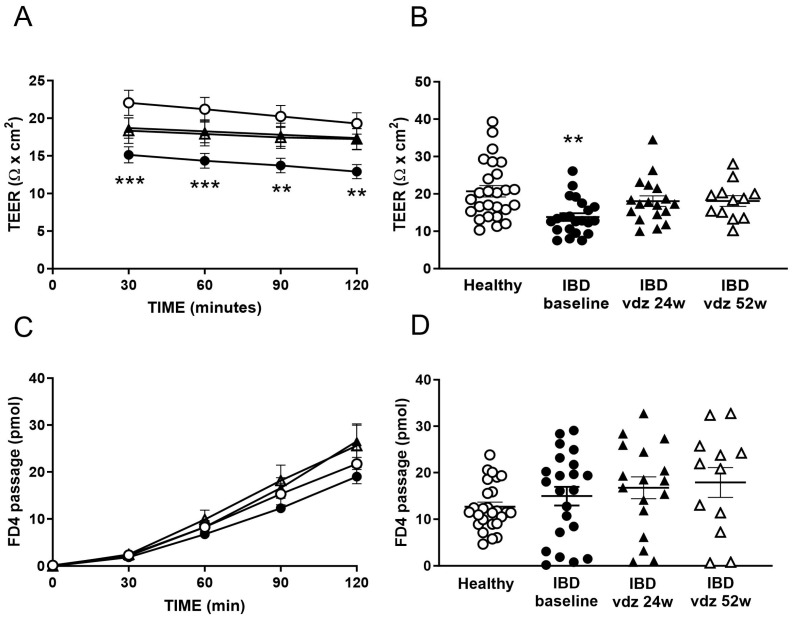
Epithelial barrier function in HC and IBD patients before and after 24 weeks and 52 weeks of treatment with vedolizumab (VDZ). Transepithelial electrical resistance (TEER) at the time points 30, 60, 90 and 120 min (**A**), and as the average over 30–120 min (**B**). Paracellular passage of FD4 at the time points 30, 60, 90 and 120 min (**C**), and as the average over 60–120 min (**D**). Data are mean ± standard deviation (SD). In (**A**–**D**): n = 26 healthy controls, n = 22 IBD patients at baseline, n = 18 IBD patients after 24 weeks of VDZ treatment, n = 12 IBD patients after 52 weeks of VDZ treatment. One-way ANOVA in (**B**,**D**) followed by Dunn’s multiple comparisons test, and two-way ANOVA in (**A**,**C**) followed by Sidak’s multiple comparisons test. ** *p* < 0.01 vs. healthy controls, *** *p* < 0.001 vs. healthy controls.

At baseline, the colonic epithelium of IBD patients showed a lower TEER than HC (13.80 ± 1.03 Ω × cm^2^ vs. 20.70 ± 1.52 Ω × cm^2^, respectively, *t*-test *p* < 0.001, Figure 2B), indicating the presence of an increased epithelial permeability to ions. This increased ionic permeability was stable and consistent during all the time points assessed (Figure 2A, ANOVA *p* < 0.01). In contrast, the paracellular permeability to FD4 did not significantly differ between patients and HC (14.12 ± 2.11 pmols vs. 12.67 ± 0.98 pmols, respectively, *p* > 0.05, Figure 2C,D).

### 2.3. Clinical Outcomes of Vedolizumab Therapy

Out of the nineteen UC patients, five (26.3%) discontinued treatment by week 24 (one due to adverse events, three due to primary non-response, and one was lost at follow-up as they refused to undergo the second colonoscopy). Six UC patients dropped out from the study during the long-term follow-up period by week 52 (three were lost at follow-up, two discontinued therapy for worsening of intestinal disease, and one due to the onset of axial arthralgia). One UC patient dropped out of the study by week 48 for worsening arthralgia. Therefore, a total of 12 patients (CD: n = 4; UC: n = 8) completed the 52 weeks of follow-up.

At week 24, a clinical response was observed in 8/14 (57%) and 1/4 (25%) patients in the UC and CD groups, respectively. Additionally, 5/14 UC (35.7%) and 1/4 CD (25%) patients presented with steroid-free clinical remission.

After 52 weeks, a clinical response was reported in 62% of patients with UC, including 42% of patients who achieved steroid-free clinical remission, while two CD patients out of four reported both a steroid-free clinical response and remission.

**Figure 3:**
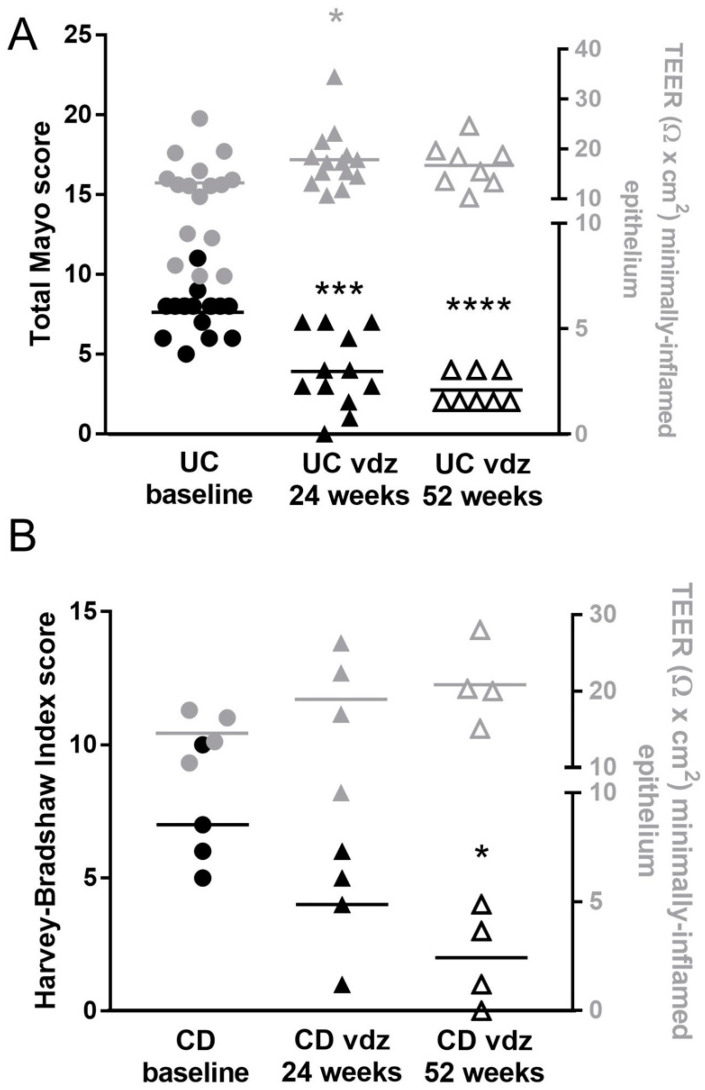
Clinical score (left axis) and TEER (right axis) in UC (**A**) and CD (**B**) patients before and after 24 weeks and 52 weeks of treatment with vedolizumab (VDZ). Data are mean values per patient at baseline and after VDZ treatment; in (**A**,**B**): n = 16 UC and n = 4 CD patients at baseline, n = 14 UC and n = 4 CD patients after 24 weeks of VDZ treatment, and n = 8 UC and n = 4 CD patients after 52 weeks of VDZ treatment. Two UC patients had no endoscopic scores after 24 weeks of VDZ treatment. For the comparison of the TEER, a one-way analysis of variance (ANOVA) followed by Holm–Sidak’s multiple comparison post hoc test was used. For the comparison of the endoscopic scores, the Kruskal–Wallis test followed by Dunn’s multiple comparison post hoc test was used. * *p* < 0.05, *** *p* < 0.001 and **** *p* < 0.0001 vs. IBD baseline (post hoc test ANOVA).

The Mayo Clinic score in UC patients significantly decreased between week 0 and week 24 [8 (6.25–8) vs. 3.5 (2.25–6.75), *p* < 0.001]. After 52 weeks, the Mayo score did not improve further when compared with week 24 [3.5 (2.25–6.75) vs. 2 (2–4), *p* > 0.05, Figure 3A]. Similar results were obtained in CD patients (Figure 3B, ANOVA *p* = 0.009) when assessing the HBI score. Fifty-two weeks of treatment significantly reduced the endoscopic score in CD patients when compared to the baseline (*p* < 0.05).

**Figure 4:**
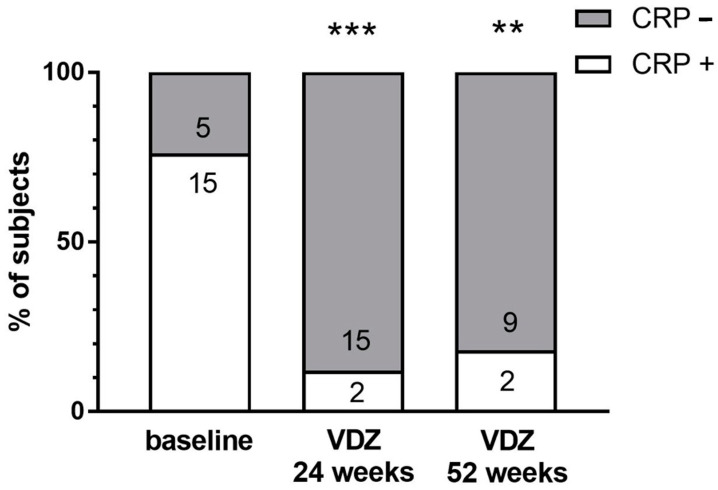
Percentage of IBD patients with a positive (+) and negative (−) C-reactive protein (CRP) test at baseline (n = 17) and after 24 weeks (n = 17) and 52 weeks (n = 11) of treatment with vedolizumab (VDZ). Numbers in the bars are the number of subjects included. The Fisher exact test was used for comparisons. ** *p* < 0.01 and *** *p* < 0.001 vs. IBD baseline.

CRP normalization was obtained at week 24 in 88.2% of patients with an elevated CRP at baseline (Figure 4) and 81% of patients at week 52. After 24 and 52 weeks of VDZ treatment, 12% and 18% of the patients still had a pathological CRP, respectively (χ^2^ = 0.0002, Figure 4).

The MES in UC patients progressively decreased between weeks 0, 24 and 52 (2.6 ± 0.5 vs. 1.9 ± 0.9 vs. 1.4 ± 1.0, *p* < 0.05 and *p* < 0.02, respectively). An endoscopic remission in UC was reported in four out of twelve (33%) patients at week 24 and in six out of nine (67%) patients at week 52. In CD patients, the Simple Endoscopic Score for Crohn Disease (SES-CD) showed an endoscopic response in three out of four patients, and a remission was achieved in one patient at week 52. In patients with clinical and endoscopic remission, histology also documented a partial remission of inflammation.

### 2.4. Vedolizumab Treatment Reduced Histological Grading of Inflammatory Activity in IBD

**Figure 5:**
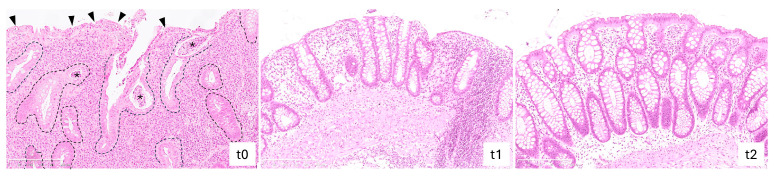
Histological grading of inflammatory activity according to the Robarts Histopathology Index (RHI) at t0, t1 and t2 time points. The t0 shows a high level of inflammation with distorted and scattered crypts (dashed lines). A larger number of neutrophilic granulocytes in the superficial epithelium (black arrowheads), in the intestinal crypts where they accumulate and form abscesses (*), and in the lamina propria were detected at t0, compared with t1 and t2. Haematoxylin and eosin (E&E) staining, O.M. X100, scale bar: 250 μm.

Following 24 (t1) and 52 (t2) weeks of treatment, a lower level of chronic inflammation and a smaller number of neutrophilic granulocytes were found both in the epithelium of the intestinal crypts and in the surrounding lamina propria, compared to the baseline (t0), with a significant difference in the RHI [6 (3.8–9.4) vs. 17 (11.4–19.8), *p* = 0.002, and 6 (3.9–7.3) vs. 17 (11.4–19.8), *p* < 0.001, respectively]. No statistically significant differences were found between t1 and t2 (Figure 5).

### 2.5. Vedolizumab Restored the Increased Permeability to Ions in IBD

**Figure 6:**
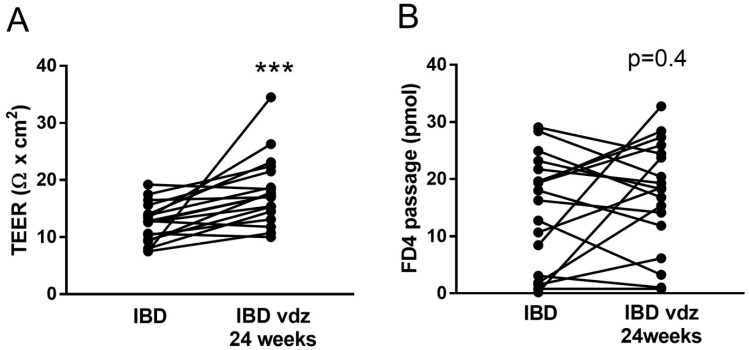
Epithelial barrier function in IBD patients after 24 weeks (t1) of treatment with vedolizumab (VDZ). Transepithelial electrical resistance (TEER) as the average over 30–120 min (**A**). Paracellular passage of FD4 as the average over 60–120 min (**B**). Data are mean values per patient before and after VDZ treatment; in (**A**,**B**): n = 18 IBD patients at baseline, and n = 18 IBD patients after 24 weeks of VDZ treatment. A paired *t*-test was used for comparison. *** *p* < 0.001 vs. IBD.

In the overall IBD group, colonic TEER significantly increased from 13.80 ± 1.03 Ω × cm^2^ to 18.06 ± 1.41 Ω × cm^2^ (n = 22 and n = 18, respectively, *t*-test *p* < 0.05) and 18.09 ± 1.44 Ω × cm^2^ (n = 12, *t*-test *p* < 0.05) after 24 and 52 weeks of treatment, respectively (Figure 2A,B and Figure 3, thereby indicating a reduced epithelial permeability to ions during therapy. In contrast, the passage of FD4 was not significantly modified after 24 weeks (14.12 ± 2.11 pmols vs. 16.75 ± 2.35 pmols, *t*-test *p* > 0.05) and 52 weeks (17.88 ± 3.21 pmols, *t*-test *p* > 0.05) of treatment (Figure 2C,D). Individual data on the TEER and FD4 passage in IBD patients at baseline and t1 are displayed in Figure 6. A paired comparison between IBD patients at the baseline and t1 showed an overall decreased permeability to ions as witnessed by an increased TEER (12.42 ± 0.80 Ω × cm^2^ to 18.06 ± 1.40 Ω × cm^2^, n = 18, *t*-test *p* < 0.001, Figure 6A) without alteration in the paracellular permeability to FD4 (14.40 ± 2.31 Ω × cm^2^ to 17.14 ± 2.25 Ω × cm^2^, n = 18, *t*-test *p* > 0.05, Figure 6B).

In parallel to the improvement in the clinical and macroscopic inflammation (total Mayo score, Figure 3A) as well as in the histological grading of inflammatory activity (Figure 5), the TEER of colonic epithelium of UC patients significantly increased after 24 weeks (from 13.20 ± 1.25 Ω × cm^2^ at baseline to 17.82 ± 1.58 Ω × cm^2^ at t1; ANOVA *p* < 0.05), remaining stable until 52 weeks (16.71 ± 4.54 Ω × cm^2^), as reported in right axis of Figure 3A. A similar trend was found in the CD patients (Figure 3B, right axis, ANOVA *p* = 0.11), although not reaching significantly differences between groups: healthy 20.89 ± 1.53 Ω × cm^2^, CD 14.50 ± 1.56 Ω × cm^2^ (n = 26 and n = 4, respectively, *t*-test *p* = 0.09).

**Figure 7:**
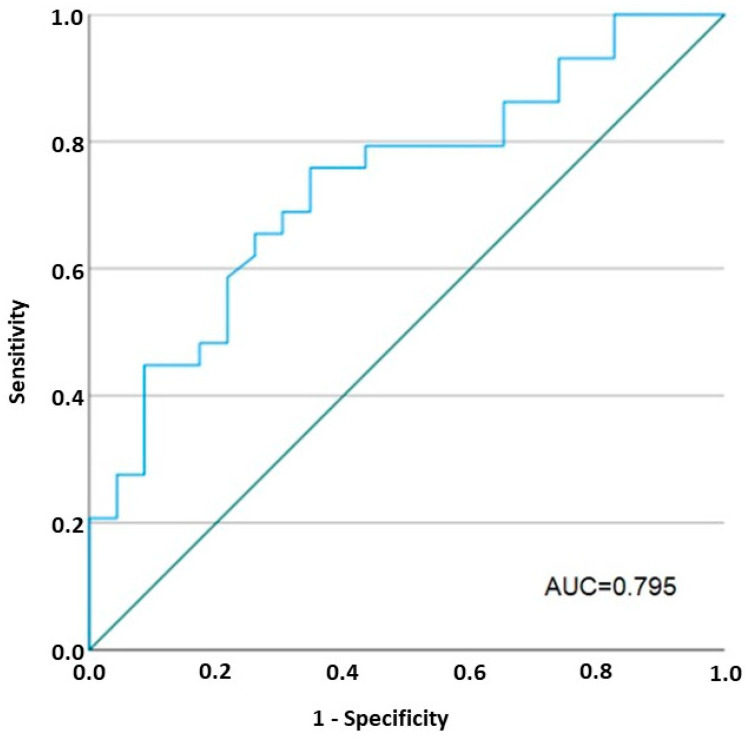
Receiver operating characteristic (ROC) curve of TEER values in patients and controls. AUC: area under the curve. The ROC curve was generated by calculating the sensitivity and 1-specificity at all possible cut-offs. A univariate regression model was created to evaluate the association between the obtained cut-off for the TEER value and clinical outcomes in all IBD patients; n = 26 healthy controls, n = 22 IBD patients at baseline, n = 18 IBD patients after 24 weeks of VDZ treatment, n = 12 IBD patients after 52 weeks of VDZ treatment. The green line is the reference line.

The AUC based on the TEER values measured in the patients and controls was 0.795 (95%CI: 0.67–0.92). A cut-off of 14.65 Ω × cm^2^ was obtained to discriminate the patients from HC (sensitivity 77%, specificity 70%) (Figure 7). According to a univariate analysis, TEER values < 14.65 Ω × cm^2^ at baseline in IBD patients were strongly associated with clinical responses to biological therapy (OR 11; CI 2–59).

## 3. Discussion

This study aimed to explore the epithelial barrier integrity ex vivo in the minimally affected colonic mucosa of IBD patients, and its relationship with clinical features. It also examined the effect of VDZ on the barrier function, which is able to decrease leukocyte trafficking and mucosal inflammation. Great attention was paid to selecting patients who were candidates for a biological treatment for moderate to severe disease, with a proper washout from antibiotics, and to include a large group of HC.

The main findings of this study are: (i) the presence of increased permeability to ions in minimally affected colonic mucosa in IBD patients compared with HC; (ii) an apparent effect of VDZ treatment in restoring the increased permeability to ions, together with a significant reduction in inflammatory activity at six and twelve-month follow-up; and (iii) a significant association between the improvement in ionic permeability (TEER) and clinical, endoscopic, and histological scores.

Gut permeability is frequently studied with in vivo methods, which cannot differentiate the precise location of the alterations and are influenced by other factors such as intestinal motility or mucosal blood flow and lack of standardization [22,23,24]. Confocal laser endomicroscopy (CLE) is an exciting method to directly assess intestinal barrier integrity during endoscopy, detecting microscopic signs of epithelial dysfunction such as fluorescein leaks or gaps [25]. These alterations could predict subsequent clinical relapse or hospitalization in IBD patients [26,27,28,29,30,31]. Nevertheless, CLE needs to be further validated. Since all techniques aimed at measuring epithelial gut integrity have some limitations [24], we used the gold standard ex vivo methodology (i.e., Ussing chambers) to study whether there is an altered colonic permeability in the minimally affected macroscopic mucosa of active IBD patients that could play a pivotal pathogenic role. We found that IBD patients have a reduced colonic TEER without changes in the paracellular passage of FD4 when compared with HC, highlighting that our patients have an overall increased colonic epithelial permeability to ions, with no effect on the paracellular permeability to large molecules.

TEER measures the net flux of all ions (cations and anions) across the epithelium, and reflects the contribution of the paracellular resistance (resistance of the TJs), but also the transcellular resistance (resistance to ions of the apical and basolateral membranes) and, finally, the subepithelial resistance [32]. The paracellular resistance exerted by the TJ structure is the major contributor to the TEER. The TJs are composed of at least two main functional pathways: the so-called pore pathway, which allows for passage of small ions and molecules with a diameter of <0.6 nm, and the leak pathway, which allows for the flux of larger ions and molecules, including FD4 [33]. The pore pathway is mainly regulated by claudins (CLDNs), and is more sensitive to a Th2 response. In contrast, the leak pathway is mainly regulated by occludin (OCLN) and the zonula occludens (ZO) family, and responds to Th1 cytokines [34]. In IBD and other inflammatory diseases, regions with severe epithelial damage allow for the non-selective flux of both ions and FD4 and large molecules, and even intact bacteria, via the unrestricted pathway [35]. We included a large cohort of 26 HC and 23 IBD patients with active inflammation. Unfortunately, only a few ex vivo studies have been published, including UC and/or CD patients, and they were carried on significantly smaller sample sizes [7,11,12,13,16,17]. Only three publications [7,11,13] studying colonic tissue with mild to moderate disease activity, including mainly UC patients, are available. In only two of them, the TEER and the paracellular permeability were assessed. In contrast to our study, these two studies show an increased paracellular passage of mannitol [7], fluorescein, and FD4 [13], together with a lower TEER. We do not know the exact reason for this discrepancy; it is possible that the small sample size of the groups included is not representative of what occurs in physiological and pathological conditions. A reduced TEER, without any alterations in the paracellular permeability of FD4, is compatible with an alteration of the pore, but not the unrestricted and leak pathways. The reason for the upregulation of the pore pathway in IBD is unknown, but several possibilities have been suggested [35].

In our series, as expected [34,36,37], VDZ proved to be effective in inducing and maintaining steroid-free remission in the majority of UC patients. Only a few studies in IBD, exclusively focused on CD patients [15,38], have investigated the effect of biological treatments on the colonic barrier function assessed ex vivo. To our knowledge, this is the first longitudinal outcome study focused on epithelial barrier function, mainly including UC patients following VDZ. At the 6 month follow-up, all patients showed a significant decrease in the ionic permeability, except for three, for whom the TEER value remained similar after treatment (Figure 6) and a clinical improvement was not observed. Similar results were observed in both UC and CD patients, reaching TEER values close to that of HC after 52 weeks of therapy. This finding is in agreement with a recent study that harnessed, in parallel groups of patients, the Ussing chamber system to demonstrate a normal ionic permeability in UC patients in remission, in the same range of HC, together with an unaltered paracellular passage to fluorescein and FD4 [13]. Interestingly, in parallel with the decrease in the ionic permeability, the histological analysis showed a reduction in the inflammatory infiltrate after 24 and 52 weeks of therapy compared to the baseline, with no significant differences between the two time points (Figure 5). It is reasonable to assume that the further clinical improvement up to 52 weeks is temporally delayed compared to the restoration of the barrier function.

Furthermore, the decrease in the paracellular permeability to ions following VDZ was strongly associated with either an improved CRP or the endoscopic and histological scores, as well as with the clinical parameters (Figure 7). According to a univariate analysis, baseline TEER values < 14.65 Ω × cm^2^ were strongly associated with, and predictive of, clinical and endoscopic response to the VDZ therapy. All these results suggest that inflammation is probably the main cause of such increased permeability, and the findings of an underlying epithelial dysfunction that could predispose subjects to develop IBD could not be confirmed here. Intriguingly, our data suggest that the TEER measurements in Ussing chambers can be used to select IBD patients with colonic involvement before VDZ treatment. Nowadays, this technique is of increasing interest, but it can only be performed in a few institutions, since it is not broadly available and requires specific training, although representing a reliable tool to measure intestinal barrier functioning and gut integrity.

Our study has the strength that a large cohort of HC and an initially large number of patients were included, which is infrequent in ex vivo studies evaluating the epithelial barrier function. However, this study has the following limitations: (1) we did not assess the transcellular passage of large molecules or bacteria that can be potentially increased in IBD [15]; and (2) in this series, a high rate of dropout, although expected in these patients, occurred during the one-year follow-up.

In conclusion, the minimally affected colonic mucosa of IBD patients has an increased colonic permeability to ions, which is reduced and maintained after 52 weeks of VDZ, consistent with a clinical, endoscopic, and histological improvement. Baseline TEER measurements could be used to discriminate whether the patients will respond to VDZ therapy. A long-term outcome study evaluating the prognostic role of the intestinal barrier healing using Ussing chambers is warranted to better identify the patients in deep remission during biological therapy.

## 4. Materials and Methods

### 4.1. Study Population

Twenty-four IBD patients (20 with UC and 4 with CD) with inadequate response, loss of response, or intolerance to at least one conventional therapy (i.e., steroids or immunosuppressants) or anti-TNF alpha (TNFα), and candidates for treatment with VDZ (Entyvio 300 mg, Takeda Pharma A/S, Taastrup, Denmark and Takeda Manufacturing GmbH, Wien, Austria), were consecutively recruited from the patients attending the Gastroenterology Unit of the University Hospital Fondazione Policlinico Campus Bio-Medico of Rome, between March 2018 and June 2020.

The inclusion criteria, were: adult IBD patients with moderate to severe disease activity, whose diagnosis has been defined by clinical, radiological, endoscopic, and histologic criteria, and candidates for treatment with VDZ. The exclusion criteria, were: non-IBD colitis (i.e., indeterminate colitis, microscopic colitis, ischemic colitis, infectious colitis, radiation colitis, diverticular disease), short bowel syndrome; use, in the previous four weeks, of antibiotics and/or drugs capable of modifying gastrointestinal motility and secretions (i.e., anticholinergics or prokinetics) or NSAIDs, pregnant or breastfeeding women, or potential pregnancy during the course of the study. Moreover, patients with CD in the upper intestinal tract, proximal to the ileum or fistulizing pattern as the only indication for biological therapy, were excluded.

The healthy control (HC) group consisted of 30 individuals, not affected by either IBD or other diseases that could potentially compromise the colonic epithelium (i.e., irritable bowel syndrome, diabetes). Fifteen subjects of a similar age to patients were recruited in each institution (Fondazione Campus Bio-Medico of Rome and TARGID, Leuven) during the same study period at screening colonoscopies for colorectal cancer, after signing an informed consent. The study was carried out by the principles of the ethical guidelines of the 1975 Declaration of Helsinki after approval by the Medical Ethics Committee of the University Hospital Fondazione Policlinico Campus Bio-Medico of Rome (14/18 PAR ComEt CBM, approved in October 2017) and UZ Leuven (S62454, approved in March 2019).

### 4.2. Clinical Evaluation

Data were collected for patient characteristics (Table 1). As expected in the management of these patients, follow-up visits were performed at baseline, after two weeks and after six weeks in the induction period, and every eight weeks during the maintenance regimen, up to the fifty-two weeks. Laboratory markers, as well as fecal tests—in order to exclude concomitant infections—were also collected at baseline and at each study visit. CRP values were considered normal if <5mg/L, according to our local laboratory cut-off. Clinical scores, such as the Harvey-Bradshaw Index (HBI) for CD and Mayo Score Index for UC, were evaluated during the initial assessment, after the sixth week, and every eight weeks until the end of the study period. Remission was defined as HBI ≤ 4 for CD, and as a partial Mayo Clinic score ≤ 1 for UC [39]. Patients with a decrease of ≥2 points in HBI compared with the baseline in CD, and of at least 2 points in the Partial Mayo score for UC patients, were considered to be clinical responders [39]. Endoscopic evaluation with multiple biopsies was performed after 24 and 52 weeks of treatment with VDZ in all patients. For UC patients, endoscopic response and remission were evaluated by assessing the Mayo endoscopic score (MES) and the simple endoscopic score for CD (SES-CD). Endoscopic response was defined as a reduction in the MES of at least 1 point and in the SES-CD of at least 50% of the baseline score; endoscopic remission was defined as a MES = 0 or 1 and SES CD ≤ 2 [39].

### 4.3. Collection of Colonic Biopsies

After providing informed consent, all patients underwent a colonoscopy to define disease activity and to collect biopsies at baseline (t0), after 24 weeks (t1), and after 52 weeks (t2) of treatment. Colonoscopies were performed after a polyethylene glycol-electrolyte solution-based preparation administered orally. All patients received conscious (midazolam ± pethidine) or deep sedation (propofol) according to our local protocol. A Jumbo forceps of 9 mm or a standard biopsy forceps were used to obtain biopsies during each procedure. Biopsies were taken in sigmoid colon one at a time. During the endoscopic procedures, depending on the disease activity, biopsies were taken from the minimally inflamed mucosa adjacent to the macroscopically affected areas (spared from more intense inflammation). During the second colonoscopy (t1) and the third colonoscopy (t2), the biopsies were taken from the same sites as the t0. For each patient, six biopsies were collected as follows: two for the histological evaluations and four for the assessment of the intestinal barrier function ex vivo. The collected biopsies were immediately transferred to the research laboratories. Ex vivo experiments were started within thirty minutes.

### 4.4. Histological Evaluation

Biopsies obtained during colonoscopy at baseline (t0), after 24 weeks (t1), and after 52 weeks (t2) of treatment were fixed in buffered formalin (11699404, Sigma-Aldrich, Merck Life Science, Milan, Italy) for 12 h and embedded in paraffin (327204, Sigma-Aldrich, Merck Life Science, Milan, Italy) with a melting point of 55–57 °C. Three- to four-micrometer sections were cut and stained with haematoxylin and eosin (H&E) stain (GHS316 and HT110216, Sigma-Aldrich, Merck Life Science, Milan, Italy) for morphological analysis. Histological grading of inflammatory activity was calculated according to the Robarts Histopathology Index (RHI) at t0, t1 and t2 time points.

### 4.5. Assessment of the Epithelial Barrier Function

The colonic epithelial integrity was analyzed in colonic biopsies mounted in the Ussing chamber system (Mussler Scientific Instruments, Aachen, Germany). The epithelial permeability to ions (transcellular and paracellular) was assessed by measuring the TEER. The paracellular permeability was assessed by using fluorescein isothiocyanate-dextran (FITC-Dextran, hereafter referred to as FD4, MW = 4 kDa). Four biopsy samples from each patient were harvested in chilled Krebs–Ringer bicarbonate solution at pH 7.4 (all chemicals were purchased from Sigma-Aldrich, St Louis, MO, USA), placed inside a modified 3 mL Ussing chamber, with an exposed area of 0.017 cm^2^, and studied as we previously described [40,41].

Briefly, the mucosal and serosal compartments of the Ussing chamber were filled with 3 mL of 10 mM mannitol and 10 mM glucose in Krebs–Ringer bicarbonate solution, respectively. The solutions were kept at 37 °C and continuously carbogenated with a mixture of O_2_/CO_2_ (95/5%). The experiment was conducted in open circuit mode. A paracellular probe (FD4, 1 mg/mL, TdBLabs, Upsala, Sweden) was then added to the mucosal compartment, and samples were collected from the serosal side every 30 min over 2 h. After sampling, fluorescence levels were measured using a fluorescence microplate reader (Tecan Infinite M200-Pro multiplate reader, Tecan, Männedorf, Switzerland). The recorded fluorescence values were converted to FD4 concentrations (pmol) using a standard curve. Since the paracellular probe takes time to accumulate on the serosal side, the samples taken at time 0 and 30 min were excluded from the statistical analysis. The average of the results obtained from the samples taken at time 60, 90 and 120 min relative to each biopsy was calculated, and the respective passage of FD4 was obtained and expressed in pmol. TEER was calculated from the voltage deflections induced by a bipolar constant-current pulse of 16 µA every 60 s and lasting 200 ms, and it was recorded every 30 min for 2 h. The average of the TEER values obtained at each collection (from 30 to 120 min) for each of the 4 biopsy samples was calculated, and the results were expressed in Ω × cm^2^, as previously described [40,41]. To ensure the proper mounting of the tissue samples in the adaptors, biopsies that showed a visual passage of FD4 and had a high passage at 30 min were excluded from the analysis. At least 3 biopsies per subject were analyzed and the mean obtained from all biopsies for each patient was used for the statistics.

### 4.6. Statistical Analysis

Data were presented either as mean ± standard error of the mean (SEM) or as median, depending on data distribution, which was checked by the Kolmogorov–Smirnov test, and the Shapiro–Wilk normality test when the sample size was small. Statistical analysis was performed using GraphPad Prism v. 6.0.

Differences between the two groups were evaluated using two-tailed unpaired *t*-tests or Mann–Whitney *U* tests for continuous variables. One-way analysis of variance (ANOVA) or Kruskal–Wallis test, followed by Holm–Sidak’s or Dunn’s multiple comparison post hoc test, were used as appropriate for multiple group comparisons. Two-way ANOVA with Bonferroni correction was used to compare the TEER and FD4 passage over time between the different groups. Categorical values were compared using the Fisher exact test. Differences were considered statistically significant with *p* < 0.05.

The ability of TEER to discriminate patients from controls was assessed using a receiver operating characteristic (ROC) analysis with calculation of the AUC, performed in all study populations. To generate the ROC curve, we calculated the sensitivity and 1-specificity at all possible cut-offs. Then, a univariate regression model was generated to evaluate the association between the obtained cut-off TEER value and clinical outcomes in all IBD patients.

## Figures and Tables

**Table 1:** Characteristics of the IBD patients and healthy controls. Characteristics of the 19 patients with active ulcerative colitis (UC), 4 patients with active Crohn’s disease (CD) and 26 healthy controls included in the study. * According to the Montreal classification [21]. Mayo endoscopic score (MES); Harvey Bradshaw Index (HBI) score; Simple Endoscopic Score-Crohn Disease (SES-CD); N/A: not applicable.

	UC	CD	Healthy Controls
**Age, media (range)**	48 (22–81)	47 (25–76)	51 (41–69)
**Gender**	7 women, 12 men	2 women, 2 men	13 women, 13 men
**Current smokers, n (%)**	4 (21%)	0 (0%)	6 (23%)
**Disease duration (mean months)**	32 (6–91)	58 (52–61)	N/A
Location and behavior *	E2 11/19 (42.1%) E3 8/19 (57.9%)	L2 B1 2/4 (50%) L3 B1 2/4 (50%)	N/A
**MES, median (range)**	2.5 (2–3)	N/A	N/A
**Total Mayo Score, median (range)**	7.57 (5–11)	N/A	N/A
**HBI score, median (range)**	N/A	7 (5–10)	N/A
**SES-CD, median (range)**	N/A	7 (4–16)	N/A
**Previous anti-inflammatory medication**	Naïve to anti-TNFα (n = 5) Refractory to anti-TNFα (n = 14)	Naïve to anti-TNFα (n = 3) Refractory to anti-TNFα (n = 1)	N/A

## Data Availability

Data are available from the authors upon reasonable request.
